# A Novel RNAi Lethality Rescue Screen to Identify Regulators of Adipogenesis

**DOI:** 10.1371/journal.pone.0037680

**Published:** 2012-06-05

**Authors:** Olivier van Beekum, Yuan Gao, Ruud Berger, Arjen Koppen, Eric Kalkhoven

**Affiliations:** 1 Department of Metabolic Diseases, Netherlands Metabolomics Centre, University Medical Centre Utrecht, Utrecht, The Netherlands; 2 Centre for Molecular and Cellular Intervention, Wilhelmina Children’s Hospital, University Medical Centre Utrecht, Utrecht, The Netherlands; Pennington Biomedical Research Center, United States of America

## Abstract

Adipogenesis, the differentiation of fibroblast-like mesenchymal stem cells into mature adipocytes, is tightly regulated by a complex cascade of transcription factors, including the nuclear receptor Peroxisome proliferator activator receptor γ (PPARγ). RNAi-mediated knock down libraries may present an atractive method for the identification of additional adipogenic factors. However, using *in vitro* adipogenesis model systems for high-throughput screening with siRNA libraries is limited since (i) differentiation is not homogeneous, but results in mixed cell populations, and (ii) the expression levels (and activity) of adipogenic regulators is highly dynamic during differentiation, indicating that the timing of RNAi-mediated knock down during differentiation may be extremely critical. Here we report a proof-of-principle for a novel RNAi screening method to identify regulators of adipogenesis that is based on lethality rescue rather than differentiation, using microRNA expression driven by a PPARγ responsive RNA polymerase II promoter. We validated this novel method through screening of a dedicated deubiquitinase knock down library, resulting in the identification of UCHL3 as an essential deubiquitinase in adipogenesis. This system therefore enables the identification of novel genes regulating PPARγ-mediated adipogenesis in a high-throughput setting.

## Introduction

The relationship between obesity and its complications, such as type 2 diabetes and cardiovascular diseases, has firmly established adipose tissue as a key regulator of glucose and lipid metabolism [1]. Adipose tissue regulates metabolism through at least two different mechanisms: the storage of lipids (as triglycerides) and the secretion of so-called adipokines, which function in an endocrine or paracrine fashion. Expansion of adipose tissue, as seen in obese individuals, not only affects the storage of lipids as triglycerides in lipid droplets, but also results in qualitative and quantitative changes in a number of adipokines [2]. The amount of mature adipocytes is largely determined by the differentiation of fibroblast-like mesenchymal stem cells into adipocytes, a process called adipogenesis [1], [3]. One of the best-established model systems for adipogenesis is the mouse 3T3-L1 cell line, which can efficiently be differentiated into mature adipocytes by hormonal stimulation under experimental conditions [4], [5]. Adipogenesis is regulated by a cascade of transcription factors, ultimately leading to the induction of the transcription factor Peroxisome proliferator activator receptor γ (PPARγ) [1], [6]. Several independent lines of investigation have led to the qualification of PPARγ as the master regulator of adipogenesis. For example, *in vitro* differentiation of fibroblasts into mature adipocytes can be induced by introduction of PPARγ [7]. In addition, this protein regulates a large set of “adipocyte genes”, involved in lipid and glucose metabolism, in a feed-forward loop with another transcription factor, C/EBPα [8], [9]. Furthermore, PPARγ ^−/−^ mice are severely lipodystrophic, while PPARγ ^+/−^ mice have reduced amounts of adipose tissue [10], [11], [12], [13]. PPARγ is also essential for the maintenance of adipose tissue, since conditional knock-out of the *Pparg* gene resulted in reduced *in vivo* survival of mature adipocytes [14]. Finally, human Familial partial lipodystrophy subtype 3 (FPLD3, MIM 604367) patients, harbouring heterozygous mutations in the *PPARG* gene, are characterized by aberrant fat distribution and metabolic disturbances, including insulin resistance and dyslipidemia [15]. Besides PPARγ, other proteins are also essential for adipogenesis, including transcription factors (e.g. KLF5 [16]), transcriptional co-regulators (e.g. TRAP220/Med1 [17], Tip60 [18], TLE3 [19] and TRIP3 [20]) and lipid droplet proteins (e.g. CIDEC/Fsp27 [21]). These essential adipogenic factors have been identified through various means, such as *in vivo* studies [16], [22], yeast 2-hybrid screening [17], peptide interaction assays [20], cDNA library high-throughput-screening [19] and co-immunoprecipitations followed by mass spectrometry [18]. Furthermore, phenotypic screening resulted in the identification of the small molecules harmine and phenamil as novel PPARγ-targeting compounds [23], [24].

Since the discovery of siRNA as a way to specifically down-regulate gene expression, a broad spectrum of siRNA libraries have been developed that are now widely used in discovering novel protein interactions and to unravel the signalling cascades playing part in various cellular processes [25]. Recent findings indicate that this technology presents an attractive alternative and complementary method for the identification of novel regulators in adipogenesis. Tang *et al.* employed a small 96 well-scale siRNA library screen to identify protein kinases involved in inhibition of insulin induced glucose uptake in fully differentiated 3T3L1 adipocytes. From this screen the integrin-linked protein kinase MAP4K4 was identified as a negative regulator of adipogenesis supressing expression of the adipogenic transcription factors C/EBPα, C/EBPβ and PPARγ [26].

However, while 3T3-L1 cells present one of the best-established models for adipogenesis, screening for novel adipogenic regulators in these cells presents several (potential) problems. First, 3T3-L1 differentiation is not homogeneous, but results in mixed cell populations, with various degrees of differentiation [27], [28], [29], [30], [31]. This, together with the genetic variation observed in these cells [32], may result in a high false discovery rate. Second, the expression levels (and activity) of adipogenic regulators is highly dynamic during differentiation [3], [6], indicating that the timing of RNAi-mediated knock down during differentiation may be extremely critical. We therefore developed a novel screening method to identify regulators of adipogenesis that makes use of a U2OS cell line stably expressing PPARγ, instead of 3T3-L1 cells. This method is primarily based on loss of blasticidin resistance via PPARγ-driven microRNA expression, with the additional advantage that overexpression of PPARγ may inhibit U2OS cell growth, as observed in other cell lines [33]. In conclusion this system uses lethality rescue rather than differentiation as a read-out method, thereby allowing the identification of novel genes regulating adipogenesis in a high-throughput fashion.

## Results

### PPARγ2 Driven miRNA Expression Results in Loss of Blasticidin Resistance

**Figure 1:**
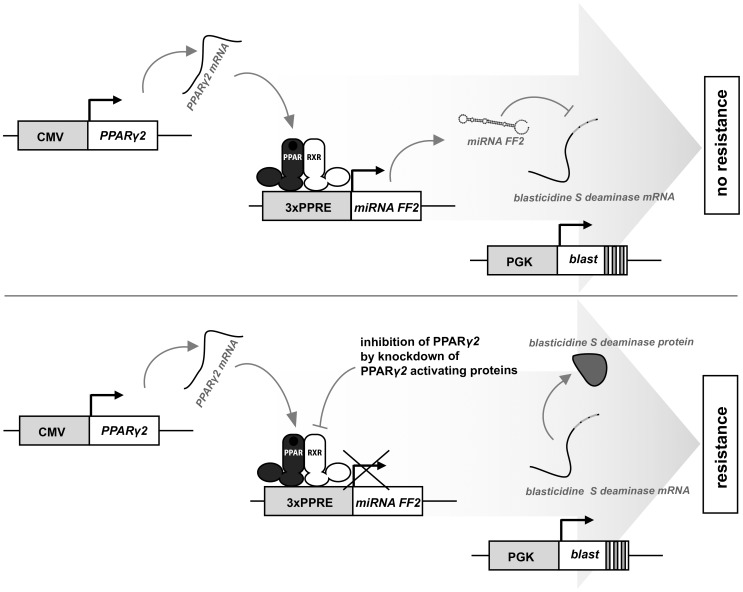
Schematic model of miRNA mediated siRNA screening. The upper panel illustrates the situation of cells with active PPARγ2-mediated gene expression. PPARγ drives miRNA FF2 expression, resulting in repression of the Blasticidin S deaminase expression cassette via the target repeat FF2 sequences present in the 3′ UTR. The lower panel depicts the situation of cells with an siRNA mediated knock-down of a PPARγ2 activating protein. Expression of the FF2 miRNA is diminished and Blasticidin S deaminase expression is no longer repressed, resulting in blasticidin resistance.

While vector-based siRNA techniques generally employ RNA polymerase III-driven expression [34], Stegmeier *et al.* recently reported efficient knock-down of gene expression from RNA polymerase II-driven miR30 miRNA-based vectors. [35]. This was achieved by replacing the region encoding the mature miR30 miRNA with sequences that encode shRNAs targeting a gene of choice. Amongst others, this system was tested using two pPRIME (potent RNA interference using microRNA expression) vectors expressing different miRNAs directed against firefly luciferase mRNA, named FF2 and FF3. Based on these vectors, we developed a novel screening method depicted here as “RNAi lethality rescue screening” (Fig. 1).

**Figure 2:**
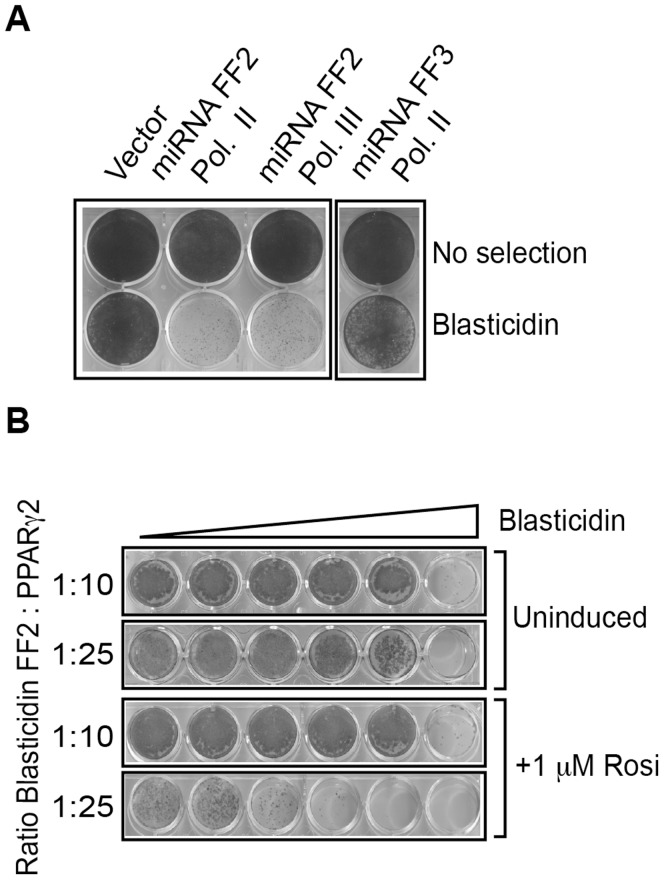
A RNA polymerase II and RNA polymerase III driven miRNA FF2 expression results in loss of Blasticidine resistance. U2OS cells expressing the Blasticidin 3×FF2 expression cassette were transduced with miRNA FF2 expressing virus and selected (50 µg/ml Blasticidin S) for 1 week. As a control either empty virus or not FF2 specific miRNA FF3 virus was used, the latter expressing miRNA not targetting the modified 3′UTR of the blasticidin S deaminase gene. B. PPARγ2 expression compared to empty vector control as detected in the stably transfected U2OS cell line also expressing the blasticidin 3×FF2 expression cassette C. U2OS cells stably transduced with 3×PPRE miRNA FF2 virus were retransduced with blasticidin 3×FF2 virus and PPARγ2 virus at a ration of 1∶10 or 1∶25 respectively. Cells transduced at a ratio of 1∶25 show loss of resistance when incubated in 1 µM rosiglitazone and increasing amounts of Blasticidin S.

For our subsequent studies we used the human U2OS osteosarcoma cell line, since these cells (i) are easy to handle and transfect, (ii) express a robust transcriptional response upon introduction of PPARγ, both on the level of reporter genes [36], [37] and endogenous target genes (data not shown), and (iii) may be growth inhibited by activation of PPARγ, a phenomenon also observed in multiple other cell lines [33], [38] which will support lethality rescue screening. We first tested whether miRNA FF2, originally directed against *luciferase* mRNA, could target a heterologous transcript. For this, 3 firefly luciferase sequences recognized by miRNA FF2 were fused to the 3′ UTR of the blasticidin resistance cassette, which encodes a Blasticidin S deaminase gene (*bsd* from *Aspergillus terreus*). Next, U2OS cells underwent retroviral transduction with a vector encoding this CMV promoter-driven blasticidin-3×FF2 cassette and stable clones were selected with Blasticidin S (Fig. 2A). To test whether constitutive miRNA FF2 expression resulted in loss of resistance, these cells were subsequently transduced with a miRNA FF2 expressing retrovirus, under control of either the RNA polymerase II-driven CMV promoter or the RNA polymerase III-driven U6 promoter. Cells expressing miRNA FF2 driven by either RNA polymerase II or RNA polymerase III showed a significant loss of blasticidin resistance (Fig. 2A). As a control, empty virus or non-specific miRNA FF3 expressing virus were used, and neither resulted in significant loss of blasticidin resistance. From these experiments we conclude that this system allows efficient expression of miRNAs by RNA polymerase II and III promoters. Furthermore, the FF2 targeting sequences, originally from the firefly luciferase gene, can be transferred to a heterologous blasticidin resistance gene, resulting in significant loss of expression of this gene when exposed to the miRNA-based FF2 vector.

### PPARγ2 Dependent miRNA Expression

Next we investigated whether the constitutively active CMV promoter, driving miRNA FF2 expression, could be replaced by a PPARγ2 responsive promoter (see also Fig. 1). We first isolated U2OS cells stably transduced with a 3×PPRE miRNA FF2 virus. The resulting U2OS 3×PPRE miRNA FF2 stable cell line underwent a second round of retroviral transduction to express Blasticidin S deaminase and PPARγ2 (Fig. 2B). When these two viruses were used at a ratio of 1∶10 respectively, activation of PPARγ by rosiglitazone had no effect on the blasticidin resitance. However, when PPARγ expression was increased by shifting the ratio 3×PPRE FF2-Blasticidin: PPARγ2 to 1∶25, a significant loss of resistance for Blasticidin S was observed in the presence of 1 µM rosiglitazone (Fig. 2C). These experiments indicate that miRNA-FF2 expression from the 3×PPRE-miRNA-FF2 vector can be induced by rosiglitazone in cells expressing sufficient amounts of PPARγ2, resulting in loss of blasticidine resistance conferred by the blasticidine-3×FF2 cassette.

**Figure 3:**
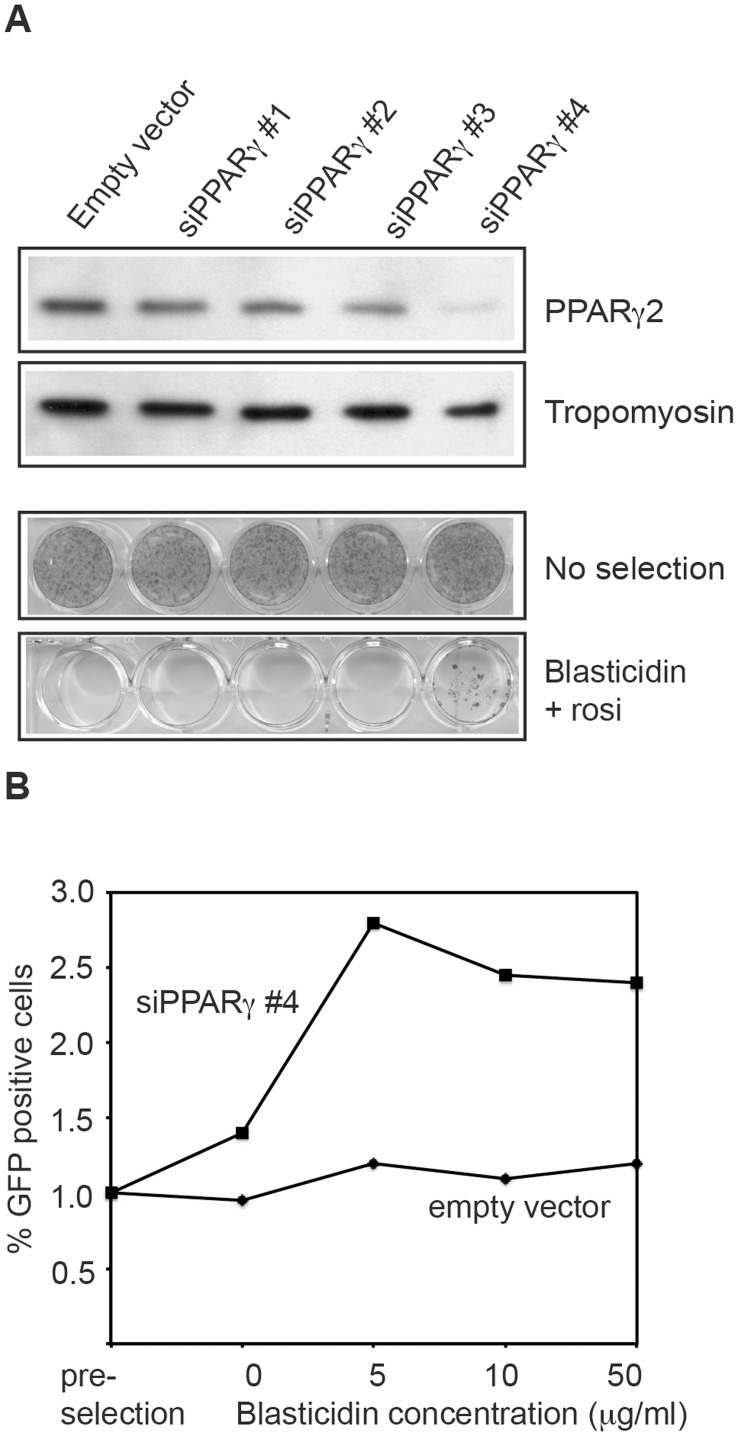
A siRNA-mediated knock down of PPARγ rescues miRNA FF2 mediated loss of blasticidin resistance. Different PPARγ specific siRNA vectors were generated and tested for their ability to rescue the loss of blasticidin resistance. The functional siRNA vector #4 rescues the miRNA FF2 induced loss of resistance. B. The U2OS 3×PPRE miRNA FF2 cells were partially rescued with PPAR#4 siRNA expressing GFP virus. The percentage of GFP positive cells was determined using FACS analysis after 1 week of blasticidin selection at various concentrations.

To verify whether the effect of PPARγ2-driven miRNA expression on loss of blasticidine resistance is indeed mediated by PPARγ2, we knocked-down PPARγ2 expression by siRNA technology. Different shRNA expression vectors directed against PPARγ were generated and transiently expressed in U2OS 3×PPRE miRNA FF2 cells. As shown in Figure 3A (upper panel), siRNA vector #4 efficiently knocked-down the expression of PPARγ, while vectors #1–3 were less efficient, as assesed by Western blotting. Next, U2OS 3×PPRE miRNA FF2 cells were transiently transfected with the different PPARγ siRNA expression vectors and incubated in the presence of rosiglitazone. After selection with Blasticidin S for 1 week in the presence of rosiglitazone (1 µM), colonies were visualized with Giemsa staining. As shown in Figure 3A (lower panel), the functional PPARγsiRNA expression vector #4 could partially rescue the loss of blasticidine resistance observed upon activation of PPARγ by rosiglitazone. Loss of blasticidin resistance was only partially rescued by considerable knock down of PPARγ activity, suggesting that the screening method filters out mainly strong activators of the PPARγ pathway. The 3 shRNA vectors which gave no efficient knock-down of PPARγ expression (#1, #2 and #3) also failed to rescue blasticidine resistance (Fig. 3A), indicating that the loss of blasticidin resistance in the presence of rosiglitazone shown in Figure 2B is indeed mediated by PPARγ. To corroborate these findings, FACS analysis was performed on the same cells with different concentrations of Blasticidin. For this we developed a retroviral GFP vector expressing the functional siRNA #4 directed against PPARγ2 described above. U2OS 3×PPRE miRNA FF2 cells were stably transduced and selected at different concentrations of Blasticidin S for 1 week. The percentage of GFP positive cells was determined by FACS analysis. The percentage of GFP postive cells after Blasticidin S selection increased approximately three fold in case of siRNA induced PPARγ2 knock down while it remained unchanged in empty GFP vector transduced cells (Fig. 3B). Taken together, these experiments indicate that the loss of resistance in U2OS 3×PPRE miRNA FF2 induced by rosiglitazone is dependent on PPARγ2 expression.

### RNAi Lethality Rescue Screening Identifies UCHL3 as a Regulator of Adipogenesis

**Figure 4:**
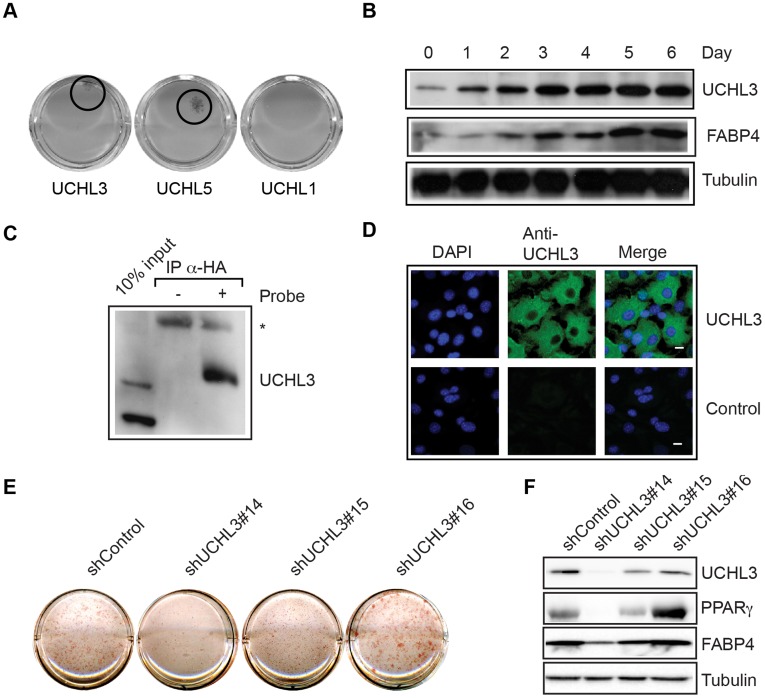
A, Small siRNA DUB screen perfomed in U2OS 3×PPRE miRNA FF2 cells. A partial siRNA library against 24 different deubiquitinating enzymes was tested for the ability to rescue the miRNA FF2 induced loss of Blasticidin resistance. Knock down of UCHL3 and UCHL5, but not UCHL1, resulted in colony formation. B. UCHL3 expression increases during 3T3-L1 adipocyte differentiation. Mouse 3T3L1 preadipocytes were differentiated into mature adipocytes and samples were taken at different time points during differentiation. Protein expression levels of UCHL3 were determined by Western blot analysis. As control for differentiation Fabp4 protein levels were analysed. C. UCHL3 activity in 3T3-L1 adipocytes. Cell lysates of differentiated 3T3-L1 cells (day 6) were incubated with or without HA-Ub probe, DUB activities were immunprecipitated (anti-HA agarose) and UCHL3 was detected by Western blotting. Note the difference in mobility between unmodified UCHL3 (input lane) and UCHL3 covalently bound to the DUB probe. An aspecific band is indicated (*). D. Localization of UCHL3 in differentiated 3T3-L1 cells. Nuclei were stained with DAPI, UCHL3 was visualized by indirect immunofluorescence. Merged pictures demonstrate the predominant cytoplasmic localization of UCHL3. As a control, the primary antibody was ommited. Bar, 10 µm. E. 3T3-L1 cells, stably transduced with lentiviral constructs expressing short hairpin RNAs directed against UCHL3 or control shRNA, were subjected to differentiation conditions. At day 10 of differentiation, cells were fixed and stained for triglycerides using Oil-red-O. Pictures are representative for three independent experiments. F. 3T3-L1 cells were stably transduced with either control or UCHL3 shRNA and differentiated as described under E. Cell lysates were subjected to Western blot analysis, using antibodies against UCHL3, PPARγ, FABP4 and tubulin.

Post-translational modifications (PTM), such as phosphorylation, acetylation, sumoylation and ubiquitination, can regulate the transcriptional output of adipogenic transcription factors like C/EBPα and PPARγ (reviewed in [39], [40]). Since the role of deubiquitylating enzymes in adipogenesis has not been studied extensively yet, we used an shRNA library that targets deubiquitinating enzymes (DUBs) [41] to validate our screening system. Four shRNA knockdown vectors against each DUB were pooled into 24 sets, where each set targets a single DUB transcript (see Table S1). The U2OS 3×PPRE miRNA FF2 cells were selected by Blasticidin S in the presence of 1 µM rosiglitazone after siRNA vector electroporation. After 3 weeks of culture, colony formation was only observed when the expression of Ubiquitin Carboxyl-terminal Hydrolase isozyme L3 (UCHL3) or UCHL5 was reduced, while for example knock down of the closely related UCHL1 enzyme did not result in colony formation (Fig. 4A).

To further investigate the potential role of UCHL3 in adipogenesis, we first examined its protein expression and localization in 3T3-L1 cells. As is shown in Figure 4B, the expression levels of UCHL3 increased during 3T3-L1 differentiation. Next, we examined the subcellular localisation of UCHL3 in mature 3T3-L1 adipocytes. In agreement with other cell types [42], [43], [44], UCHL3 displayed cytoplasmatic localization in 3T3-L1 adipocytes (Fig. 4D). Finally, we addressed the relevance of UCHL3 in adipogenesis using two different approaches. First, UCHL3 enzymatic activity was determined, using an HA-tagged ubiquitin probe. This probe is recognized by active DUBs, after which the reactive group attached to the probe covalently and irreversibly binds DUBs [45], [46]. Immunoprecipitation of active DUBs from mature 3T3-L1 adipocytes using anti-HA antibodies followed by Western blotting with UCHL3 antibodies showed that UCHL3 is an enzymatically active DUB in mature adipocytes (Figure 4D). To address the relevance of UCHL3 in adipogenesis, the expression of this protein was reduced. Adipogenesis was inhibited by lentiviral short hairpin constructs against UCHL3 (shUCHL3 #14, 15 and 16), as illustrated by triglyceride staining with Oil-red-O (Fig. 4E) and PPARγ and FABP4 expression (Fig. 4F). The degree of inhibition correlated with the reduction in UCHL3 protein expression, with shUCHL3 #14 being the most efficient and shUCHL3 #15 displaying a partial effect, and underscores the importance of UCHL3 in 3T3-L1 adipocyte differentiation. Taken together, our findings indicate that novel regulators of adipogenesis can be identified by the RNAi lethality rescue screening method described here.

## Discussion

To identify novel regulators of adipogenesis, we have developed an RNAi lethality rescue screen. This method is based on cell survival, which is accomplished through inhibition of the adipogenic transcription factor PPARγ, resulting in blasticidin resistance and possibly inhibition of PPARγ-mediated growth arrest [33]. A cell survival-based method has the advantage over, for example reporter based screening methods, that it can potentially be used for screening of pooled RNAi libraries. Validation of the RNAi lethality rescue screen described here enabled us to identify UCHL3 as a regulator of PPARγ-mediated differentiation of 3T3-L1 preadipocytes. In agreement with this, *Uchl3* ^−/−^ MEFs were very recently shown to display impaired adipocyte differentiation and lipid accumulation [47]. Morever, *Uchl3* ^−/−^ mice displayed a reduction of adipose tissue mass and were protected against high-fat diet-induced obesity and insulin resistance [47], [48]. Together with the current study, these findings strongly support a critical role for UCHL3 in adipogenesis, both *in vitro* and *in vivo*. Interestingly, the critical role of UCHL3 in adipogenesis may not be limited to its ubiquitin hydrolase activity, as this protein has dual specificity for ubiquitin and Nedd8, a ubiquitin-like protein *in vitro* [49], [50] and *in vivo* [51]. Like other post-translational modifications, neddylation can alter substrate function and activity by inducing conformational changes, or by preventing or inducing protein-protein interactions [52]. The critical deubiquitination and/or deneddylation substrates of UCHL3 in adipogenesis remain to be established. Suzuki *et al.* have shown that UCHL3 enhances insulin signalling in (pre)adipocytes, but UCHL3 is unlikely to target critical components of insulin signalling like the insulin receptor, the IGF-I receptor, IRS-1 and Grb10 [47]. Interestingly, these authors also observed impaired expression of the late adipogenic genes *fabp4*, *adiponectin* and *srebp1c*, three direct target genes of PPARγ [8], [53], [54], in *Uchl3* ^−/−^ MEFs and epididymal WAT of *Uchl3* ^−/−^ mice [47]. Ectopic expression of UCHL3, but not the catalytic mutant C95S, restored expression of these genes in *Uchl3* ^−/−^ MEFs [47]. Together with the screening method presented here, which is based on PPARγ activity, these findings suggest that PPARγ may be a direct target for UCHL3. However, in agreement with other studies [42], [43], [44], we found UCHL3 to be localized in the cytoplasm, while PPARγ was exclusively nuclear in mature 3T3-L1 adipocytes. In addition, recombinant UCHL3 failed to deubiquitinate PPARγ *in vitro* (Figure S1 and Materials and Methods SI), indicating that UCHL3 modulates PPARγ activity by an indirect mechanism, either through its ubiquitin hydrolase or its deneddylase activity.

Taken together, we have developed and validated a novel RNAi screen, based on PPARγ induced growth arrest and loss of resistance. This screening method was validated on a small scale using an shRNA library targeting different deubiquitinating enzymes. From these initial screens we identified UCHL3 as a regulator of adipogenesis. In the future PPARγ-mediated RNAi lethality rescue screening may allow high throughput screening of pooled RNAi libraries. The modules of this system can also readily be exchanged for other reporters and/or expression vectors, including heterologous reporters in combination with fusions of protein domains (Gal4, LexA) (Fig. 1). Modified forms of this lethality rescue screening method may therefore present a more generic tool to identify regulatory proteins in fundamental cellular processes.

## Materials and Methods

### Materials

RNAiMax was purchased from Invitrogen (Carslbad, USA). The following antibodies were used: anti-PPARγ (sc-7273), Santa Cruz Biotechnologies; anti-UCHL3 (3525), Cell Signalling Technology; anti-tubulin (ab6046) Abcam; anti-rabbit-HRP (111035144) and anti-mouse-HRP (115035146), Jackson Immunoresearch Laboratories Inc.; mouse anti-rabbit Alexa488, Invitrogen. The vinyl methyl ester HA-DUB probe (HAUbVME) was generated and used as described [45].

### Plasmids

The retroviral expression plasmid pMSCV-mPPARγ2 (puro) was a kind gift from Dr. B.M. Spiegelman [55]. The pPrime miRNA expression CMV miRNA FF2 and FF3 vectors were a kind gift from the Elledge lab and used for subsequent cloning [35]. Target sequence with a mismatch at the first base for FF2; cCCGCCTGAAGTCTCTGATTAA and for FF3; aGCTCCCGCTGAATTGGAATCC. A *Bgl*II*-Hind*III fragment encompassing the 3×PPRE-TK promoter was digested from the 3×PPRE-TK-luciferase reporter and subcloned into pLNCX Δ*Cla*I. The miRNA FF2 cassette was digested from pPrime using *Cla*I, *Not*I and subcloned behind the 3×PPRE-TK promoter to generate the pLNCX 3×PPRE FF2miRNA retroviral vector. A SV40 polyA signal oligo was ligated at the 3′ end of the FF2 cassette, using a *Cla*I site. All recombinant DNA work was performed according to standard procedures [56]. All mutations were generated by Quickchange mutagenesis (Stratagene) and verified by sequencing.

### Cell culture and Differentiation Assays

The human osteosarcoma cell line U2OS (ATCC, Manassas, VA) and the Phoenix amphotropic packaging cell line (Allel Biotechnology, San Diego, CA) were maintained in DMEM Glutamax (Dulbecco) containing 10% foetal calf serum (Gibco Life Technologies), 100 µg/ml penicillin and 100 µg/ml streptomycin (Gibco Life Technologies). Electroporations of U2OS cells were performed with the Biorad Genepulser Xcell using 2 µg plasmid DNA and 100 ul cell suspension in electroporation buffer (2 mM Hepes pH 7.2, 15 mM K_2_PO_4_/KH_2_PO_4_, 250 mM Manitol and 1 mM MgCl_2_ pH 7.2) per electroporation, with two times 8 pulses at 140 V, 1.5 msec burst duration at intervals of 1.5 s.

After 1 week of Blasticidin S selection at different concentrations (0, 5, 10 and 50 µg/ml) cells were either fixed and subjected to Giemsa staining or trypsinized and subsequently used for FACS analysis. After brief centrifugation cells were resuspended in ice cold PBS. The percentage of GFP positive cells of the total cell population was determined after counting 10.000 cells using a FACScan (Becton Dickinson, Biosciences).

Lentivirusses for transduction of UCHL3 short hairpin constructs were generated in HEK293T cells using the Mission® system (Sigma-Aldrich). As control the pLKO.1-puro Non-Mammalian shRNA Control plasmid (SHC002) was used. After lentiviral transduction, shRNA expressing cells were selected by puromycin selection. Differentiation of shRNA expressing 3T3-L1 cells, Oil-red-O staining and Western blotting were performed as described [18], [20].

### Immunofluorescence

For immunofluorescence staining, 3T3-L1 cells were plated on glass coverslips. Cells were differentiated for 5 days, fixed with 4% paraformaldehyde for 20 min RT and permeabilized in PBS supplemented with 0.5% Triton for 10 min. After 30 min incubation in blocking buffer, cells were stained with primary antibodies for 2 h at RT, then incubated with secondary fluorochrome-conjugated antibodies. After several washes, coverslips were mounted in Dabco-DAPI and analyzed with an LSM710 Met confocal microscope (Carl Zeiss, Jena, Germany).

## Supporting Information

Figure S1 **UCHL3 fails to deubiquitinate PPAR**γ ***in vitro*** **.** HEK293T cells were transfected with HA-tagged PPARγ expression construct together with histidine-tagged ubiquitin (His-ubi) expression construct and treated with MG132 (3 µM). Ubiquitinated proteins were isolated by Ni-NTA precipitation, eluted from the Ni-NTA beads with imidazole and incubated with recombinant UCHL3 enzyme. Ubiquitinated PPARγ was detected by Western blotting (anti-HA antibody).
(DOCX)

Table S1 **Deubiquitinases tested in screening procedure.**
(DOCX)

Materials and Methods SI (DOCX)
