# Supplementary material for: A Novel RNAi Lethality Rescue Screen to Identify Regulators of Adipogenesis
Source: PLoS One. 2012 Jun 5;7(6):e37680. doi: 10.1371/journal.pone.0037680 (PMC3367974; doi:10.1371/journal.pone.0037680)
Supplement: Figure S1 — UCHL3 fails to deubiquitinate PPARγ in vitro . HEK293T cells were transfected with HA-tagged PPARγ expression construct together with histidine-tagged ubiquitin (His-ubi) expression construct and treated with MG132 (3 µM). Ubiquitinated proteins were isolated by Ni-NTA precipitation, eluted from the Ni-NTA beads with imidazole and incubated with recombinant UCHL3 enzyme. Ubiquitinated PPARγ was detected by Western blotting (anti-HA antibody). (DOCX) [file pone.0037680.s001.docx]

**Figure S1**

**
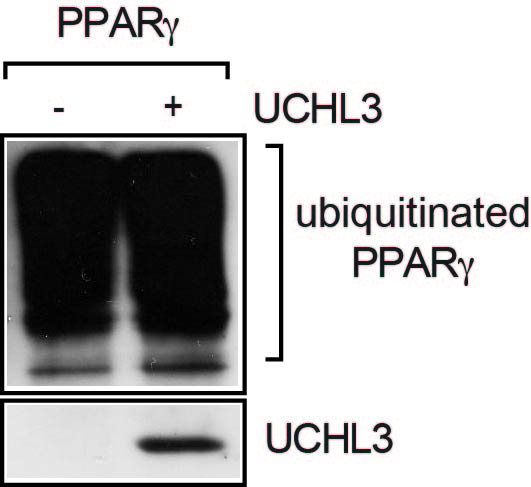
**

Figure S1 UCHL3 fails to deubiquitinate PPARγ *in vitro*. HEK293T cells were transfected with HA-tagged PPARγ expression construct together with histidine-tagged ubiquitin (His-ubi) expression construct and treated with MG132 (3μM). Ubiquitinated proteins were isolated by Ni-NTA precipitation, eluted from the Ni-NTA beads with imidazole and incubated with recombinant UCHL3 enzyme. Ubiquitinated PPARγ was detected by Western blotting (anti-HA antibody).
