# Supplementary material for: A Novel RNAi Lethality Rescue Screen to Identify Regulators of Adipogenesis
Source: PLoS One. 2012 Jun 5;7(6):e37680. doi: 10.1371/journal.pone.0037680 (PMC3367974; doi:10.1371/journal.pone.0037680)
Supplement: Table S1 — Deubiquitinases tested in screening procedure. (DOCX) [file pone.0037680.s002.docx]

**Table S1 Deubiquitinases tested in screening procedure**

|  | HGNC name | Alternative name | Ensembl gene ID | Gene ID |
| --- | --- | --- | --- | --- |
| 1 | USP12* |  | ENSG00000152484 | 219333 |
| 2 | USP11 |  | ENSG00000102226 | 8237 |
| 3 | USP10 |  | ENSG00000103194 | 9100 |
| 4 | USP7 |  | ENSG00000171616 | 7874 |
| 5 | USP8 |  | ENSG00000138592 | 9101 |
| 6 | USP40 |  | ENSG00000085982 | 55230 |
| 7 | PAN2 | USP52 | ENSG00000135473 | 9924 |
| 8 | USP46 |  | ENSG00000109189 | 64854 |
| 9 | USP31 |  | ENSG00000103404 | 57478 |
| 10 | USP48 |  | ENSG00000090686 | 84196 |
| 11 | USP30 |  | ENSG00000135093 | 84749 |
| 12 | UCHL5# |  | ENSG00000116750 | 51377 |
| 13 | UCHL3 |  | ENSG00000118939 | 7347 |
| 14 | UCHL1 |  | ENSG00000154277 | 7345 |
| 15 | USP38 |  | ENSG00000170185 | 84640 |
| 16 | USP44 |  | ENSG00000136014 | 84101 |
| 17 | USP39 |  | ENSG00000168883 | 10713 |
| 18 | USP54 |  | ENSG00000166348 | 159195 |
| 19 | USP36 |  | ENSG00000055483 | 57602 |
| 20 | USP42 |  | ENSG00000106346 | 76800 |
| 21 | USP18 |  | ENSG00000099996 | 24110 |
| 22 | USP20 |  | ENSG00000136878 | 10868 |
| 23 | USP24 |  | ENSG00000162402 | 23358 |
| 24 | USP37 |  | ENSG00000135913 | 57695 |
|  |  |  |  |  |
|  | * Ubiquitin specific protease |  |  |  |
|  | #UBIQUITIN CARBOXYL-TERMINAL HYDROLASE |  |  |  |
