# Supplementary material for: A Novel RNAi Lethality Rescue Screen to Identify Regulators of Adipogenesis
Source: PLoS One. 2012 Jun 5;7(6):e37680. doi: 10.1371/journal.pone.0037680 (PMC3367974; doi:10.1371/journal.pone.0037680)
Supplement: Materials and Methods SI — (DOCX) [file pone.0037680.s003.docx]

**Materials and Methods SI**

**Deubiquitination assays**

For *in vitro* (de)ubiquitination assays, cells were transfected with HA-PPARγ and His-ubi expression plasmids. Twenty-four hours after transfection, cells were incubated o/n with MG132 (3 μM), lysed and his-tagged proteins were isolated as described [[1](#_ENREF_1),[2](#_ENREF_2)]. Recombinant human UCHL3 (E-325; Boston Biochem, Boston, MA) was added to the substrates for 1 hour at 37ºC in deubiquitination buffer (50 mM Tris PH 7.4, 150 mM NaCl, 10 mM DTT, 5 mM MgCl_2_). Reactions were stopped by adding 2xSDS sample buffer and analysed by Western blotting.

**References**

1. Stad R, Little NA, Xirodimas DP, Frenk R, Van Der Eb AJ, et al. (2001) Mdmx stabilizes p53 and Mdm2 via two distinct mechanisms. EMBO Rep 2: 1029-1034.

2. Meulmeester E, Maurice MM, Boutell C, Teunisse AF, Ovaa H, et al. (2005) Loss of HAUSP-mediated deubiquitination contributes to DNA damage-induced destabilization of Hdmx and Hdm2. MolCell 18: 565-576.
